# Zebrafish Model-Based Assessment of Indoxyl Sulfate-Induced Oxidative Stress and Its Impact on Renal and Cardiac Development

**DOI:** 10.3390/antiox11020400

**Published:** 2022-02-16

**Authors:** Paul Wei-Hua Tang, Ping-Hsun Wu, Yi-Ting Lin, Chen-Hao Chiu, Tien-Li Cheng, Wen-Hui Guan, Hugo You-Hsien Lin, Kun-Tai Lee, Yau-Hung Chen, Chien-Chih Chiu, Wangta Liu

**Affiliations:** 1 Department of Internal Medicine, Taipei Veterans General Hospital Yuli Branch, Hualien 981, Taiwan; africapaul12@gmai.com; 2 Faculty of Medicine, School of Medicine, National Yang Ming Chiao Tong University, Taipei 112, Taiwan; 3 Division of Nephrology, Department of Internal Medicine, Kaohsiung Medical University Hospital, Kaohsiung Medical University, Kaohsiung 807, Taiwan; 970392@mail.kmuh.org.tw (P.-H.W.); and yukenlin@gmail.com (H.Y.-H.L.); 4 Department of Family Medicine, Kaohsiung Medical University Hospital, Kaohsiung 807, Taiwan; 960254@mail.kmuh.org.tw; 5 Department of Biotechnology, Kaohsiung Medical University, Kaohsiung 807, Taiwan; plusbeetle10@gmail.com (C.-H.C.); sm89632@gmail.com (T.-L.C.); wendy006030@gmail.com (W.-H.G.); cchiu@kmu.edu.tw (C.-C.C.); 6 Department of Internal Medicine, Kaohsiung Municipal Ta-Tung Hospital, Kaohsiung 801, Taiwan; 7 Department of Medicine, College of Medicine, Kaohsiung Medical University, Kaohsiung 807, Taiwan; 8 Cardiovascular Center, Kaohsiung Medical University Hospital, Kaohsiung 807, Taiwan; kuntai.lee@gmail.com; 9 Department of Chemistry, Tamkang University, Tamsui, New Taipei City 251, Taiwan; yauhung@mail.tku.edu.tw; 10 Department of Medical Research, Kaohsiung Medical University Hospital, Kaohsiung 807, Taiwan; 11 Center for Cancer Research, Kaohsiung Medical University, Kaohsiung 807, Taiwan

**Keywords:** indoxyl sulfate (IS), mitogen-activated protein kinase (MAPK) pathway, ROS, inflammation, zebrafish model

## Abstract

Kidney disease patients may have concurrent chronic kidney disease-associated mineral bone disorder and hypertension. Cardiovascular disease (CVD) and neuropathy occur due to kidney failure-induced accumulation of uremic toxins in the body. Indoxyl sulfate (IS), a product of indole metabolism in the liver, is produced from tryptophan by the intestinal flora and is ultimately excreted through the kidneys. Hemodialysis helps renal failure patients eliminate many nephrotoxins, except for IS, which leads to a poor prognosis. Although the impacts of IS on cardiac and renal development have been well documented using mouse and rat models, other model organisms, such as zebrafish, have rarely been studied. The zebrafish genome shares at least 70% similarity with the human genome; therefore, zebrafish are ideal model organisms for studying vertebrate development, including renal development. In this study, we aimed to investigate the impact of IS on the development of zebrafish embryos, especially cardiac and renal development. At 24 h postfertilization (hpf), zebrafish were exposed to IS at concentrations ranging from 2.5 to 10 mM. IS reduced survival and the hatching rate, caused cardiac edema, increased mortality, and shortened the body length of zebrafish embryos. In addition, IS decreased heart rates and renal function. IS affected zebrafish development via the ROS and MAPK pathways, which subsequently led to inflammation in the embryos. The results suggest that IS interferes with cardiac and renal development in zebrafish embryos, providing new evidence about the toxicity of IS to aquatic organisms and new insights for the assessment of human health risks. Accordingly, we suggest that zebrafish studies can ideally complement mouse model studies to allow the simultaneous and comprehensive investigation of the physiological impacts of uremic endotheliotoxins, such as IS, on cardiac and renal development.

## 1. Introduction

Chronic kidney disease (CKD) is one of the leading chronic diseases, and kidney failure affects entire body systems. CKD has been reported to cause heart-related diseases, which are the leading causes of death in patients on dialysis [1] and may also have concurrent effects on many organs. The uremic toxin indoxyl sulfate (IS) that is produced by tryptophan metabolism accumulates during CKD. IS plays a role in cardiovascular pathogenesis and is classified as a uremic endotheliotoxin because it causes endothelial dysfunction that is associated with cardiovascular morbidity and mortality in patients with CKD. The connection between plasma IS levels and cardiovascular morbidity and death in CKD patients has been confirmed in several investigations [2]. Unfortunately, although dialysis removes most of the nephrotoxin from the blood, IS bound to albumin causes toxic effects; the resulting combination is difficult to remove and accumulates in the human body [3].

Reactive oxygen species (ROS) include superoxide anions (O_2_^•−^), hydroxyl radicals (^•^OH), and hydrogen peroxide (H_2_O_2_). Dramatic accumulation of ROS can cause proliferation arrest and induce apoptosis of cells [4]. ROS accumulation also affects NO production and impairs neovascularization. In vitro, it has been shown to cause endothelial toxicity [2]. Exposure of endothelial cells to IS increases the formation of ROS [5].

Previous studies have shown that IS causes the death of cortical neurons and hippocampal neurons in the central nervous system and increases ROS levels in astrocytes. Moreover, primary culture with a mixture of astrocytes and microglia results in increased ROS levels and the release of the inflammatory factors TNF-α and IL-6 [6]. However, many studies evaluating IS toxicity in animals have been limited to mouse models. In addition, the protocols and animal models used in these studies, including models with normal function, subtotal nephrectomy, and adenine diet feeding, depend on the administration of IS through intraperitoneal or oral routes to assess renal injury [7].

Although the pathological effects of IS have been established using mouse in vivo models, zebrafish (*Danio rerio*)-based models have been shown to be suitable for high-throughput drug screening and toxicity testing in 96-well plates due to the short growth period and transparent embryos of zebrafish [8]. Many studies have recently used zebrafish as model organisms to develop new high-throughput screening platforms [9]. Zebrafish-based in vivo models can be used to assess the physiological and developmental impacts of IS. Zebrafish are tropical freshwater fish that have been widely used in research in recent years as model organisms because they share 70% orthologous gene similarity with humans [10]. Zebrafish models are used for drug screening [11]. The organ systems of zebrafish are highly similar to those of humans, and the toxicity of drugs in zebrafish is similar to that in mammals. Zebrafish are becoming widely used biological models for chemical toxicity screening tests [12]. Furthermore, zebrafish have a central nervous system similar to that of humans, and the two species have high degrees of physiological and genetic homology [13]. Many biomedical studies have selected zebrafish as experimental animal models to understand human disease patterns [14]. A database of different transgenic zebrafish and zebrafish mutants with different disease patterns has been created [15]. Although many studies have reported the use of zebrafish to establish high-throughput screening platforms, such as platforms for angiogenesis and developmental toxicity screening [9,11,16,17], a zebrafish model for studying the developmental effects of IS on zebrafish has not been established.

Renal and cardiac development occurs in the early stage of embryonic development in both zebrafish and mice [18,19]. In addition, primary heart tube formation and looping occur early in the development of the heart in both zebrafish and mice. Notably [18], zebrafish embryos are transparent, and developmental changes in the heart and kidneys can be easily observed without sacrificing the embryos. In contrast, mice are viviparous animals; thus, it is necessary to sacrifice both maternal mice and embryos to evaluate the developmental changes in the hearts and kidneys of the embryos, which is contrary to the principle of the “3 Rs” (Replacement, Reduction, and Refinement). As a result, zebrafish can be used as ideal animal models for investigating the early stage of cardiac development (with loop formation as the boundary). Therefore, the two animal models mentioned above can complement each other.

This study investigated the toxic effects of IS on cardiac and renal development in zebrafish embryos and examined the mechanisms involved.

## 2. Materials and Methods

### 2.1. Cell Culture

HEK293T human embryonic kidney cells (gifts from Dr. Jin-Ching Lee, National Sun Yat-sen University, Kaohsiung, Taiwan) were incubated in a 100 mm^2^ petri dish in 3:2 DMEM:F-12 medium supplemented with 2 mM glutamine, 8% fetal bovine serum, 100 units/mL penicillin and 100 μg/mL streptomycin (Gibco, Gaithersburg, MD, USA) in a 5% CO_2_ humidified atmosphere at 37 °C.

### 2.2. Assessment of Cell Viability

A colorimetric 3-(4,5-dimethylthiazol-2-yl)-5-(3-carboxymethonyphenol)-2-(sulfophenyl)-2H-tetrazolium (MTS, Promega, Madison, WI, USA) test was used to determine cell viability. In a 96-well plate, 5 × 10^3^ cells were seeded, and the cells were administered the indicated concentrations of IS and incubated for 24 h and 48 h. MTS and phenazine methyl sulfate (PMS, Sigma–Aldrich, St. Louis, MO, USA) were added to each well to achieve final concentrations of 80 g/mL and 7.3 g/mL, respectively, and the cells were incubated further. Next, the absorbance was measured at 490 nm using a microplate reader (MTX Lab Systems, Inc., Vienna, VA, USA) and used to represent the relative cell viability.

### 2.3. Colony Formation Assay

A total of 4 × 10^2^ HEK293T cells were seeded in a 12-well plate overnight and incubated with the indicated concentrations of IS for 11 days. The cells were paraformaldehyde-fixed and then Giemsa-stained. All colonies in each well of the plate were counted [20].

### 2.4. Animals and IS Exposure

The adult AB strain, *Tg(fli1:GFP)* and *Tg(wt1b:GFP)* [21] zebrafish were supplied by Academia Sinica’s Zebrafish Core Facility (TZCAS, Taipei, Taiwan) and Professor Christoph Englert. The zebrafish assays were approved by Kaohsiung Medical University (KMU), Kaohsiung, Taiwan (IACUC approval #107091). The zebrafish adults and embryos were cared for and maintained in accordance with the rules and standard protocols for animal care of the animal center of KMU [22]. The zebrafish were housed in aquaria at 28.5 °C under a 10 h dark/14 h light night/day cycle in the Zebrafish Core Facility, which was accredited by the Association for Assessment and Accreditation of Laboratory Animal Care International (AAALAC). For breeding, adult male and female fish were housed in a breeding tank at a ratio of 3 males to 2 females. The embryos were collected from the bottom of the tank and washed twice with oxygenated water. Indoxyl sulfate with potassium salt (Cayman Chemical Co., Ann Arbor, MI, USA) was dissolved in Milli-Q water as a vehicle solvent. The embryos were treated with 2.5 mM, 5 mM, or 10 mM IS at 24 h postfertilization (hpf) and examined daily.

### 2.5. Histologic Studies

Zebrafish larvae treated with 2.5 mM IS at 72 hpf were fixed in 10% formaldehyde (Nihon Shiyaku Industrial Ltd., Tokyo, Japan), soaked overnight, and then immersed in 2% agarose LE (Cyrusbioscience) in water. The specimens were sliced at a thickness of 4–5 μm and embedded in 2% agarose LE (Cyrusbioscience), and the heads of the embryos were aligned and embedded in paraffin. After deparaffinization, the tissue was stained with hematoxylin and eosin (HE, Leica) for histologic examinations.

### 2.6. ROS Assessment

At the 48 hpf time point, zebrafish embryos were transferred into a 1.5 mL centrifuge tube, 500 μL of 5 μM dihydroethidium (DHE, Invitrogen, Waltham, MA, USA) was added, and the tubes were shaken on a shaker for 40 min. The stain was then aspirated, and the embryos were washed three times with water for 10 min each time before being transferred to 3% cellulose and observed under a fluorescence microscope.

### 2.7. Analysis of the Effects of Treatment on Zebrafish Morphology and Survival

Dechorionated embryos were immobilized with 3% cellulose on a glass depression slide at 72 hpf. Using a stereomicroscope, the morphology was evaluated visually (Leica MZ10 F, Leica Microsystems Ltd., Buffalo Grove, IL, USA). Similarly, light microscopy was used to evaluate the survival of embryos at 8-h intervals up to 96 hpf. Cardiovascular contractility was used as the criterion for the survival of zebrafish embryos.

### 2.8. Renal Function Assay

The determination of renal function was performed by measuring the clearance of 10-kDa dextran labeled with tetramethylrhodamine from the cardiac area as described previously [23]. The zebrafish larvae were anesthetized with 0.04% 2-phenoxyethanol prior to the microinjection of dextran. Then, 25–50 ng of tetramethylrhodamine-labeled dextran was microinjected into the pericardial sac at 48 hpf. After microinjection, 2-phenoxyethanol was replaced with oxygenated water, and the zebrafish embryos were maintained at 28.5 °C. Each fish was imaged 4 and 24 h after injection using a Neo 5.5 sCMOS CCD (OXFORD ANDOR). The exposure time and gain were maintained constant. The average fluorescence intensity at the center of the cardiac area was assessed and reported in relative units of brightness. Ten fish were evaluated in each group (control and 2.5 mM IS).

### 2.9. Total RNA Extraction

A 1.5 mL centrifuge tube was filled with a fixed number (15–20) of zebrafish embryos treated with IS at 72 hpf, and 500 μL of TRIzol Reagent (Invitrogen, Waltham, MA, USA) was added. The embryos were ground with a grinding rod and then placed on ice for 5 min. The embryos were then centrifuged at 12,000× *g* for 10 min at 4 °C. The supernatant (approximately 500 μL) was transferred to another 1.5 mL centrifuge tube, and 100 μL of 1-bromo-3-chloropropane (Sigma, St. Louis, MO, USA) was added. The tube was shaken uniformly for 15 s and then centrifuged at 12,000× *g* for 15 min at 4 °C. At this point, the contents of the centrifuge tube were divided into three layers. Next, 250 μL of the top layer was added to a new 1.5 mL centrifuge tube, isopropanol (anReac AppliChem, Darmstadt, Germany) was added, and the tube was shaken uniformly. Ethanol (Sigma, St. Louis, MO, USA) was used to wash the pellet after centrifugation at 12,000× *g* for 15 min at 4 °C. After aspirating the ethanol, the pellet was centrifuged at 4 °C for 15 min. After aspirating the ethanol, the centrifuge tube containing the pellet was placed in an oven to dry the remaining ethanol for approximately 15 min. Finally, the RNA was solubilized with 20 μL of DEPC H_2_O (Protech) at 60 °C. The purity of RNA was determined by UV absorbance, and an OD260/280 ratio of approximately 2.0 was accepted.

### 2.10. Next-Generation Sequencing (NGS) Analysis

Library construction and RNA sequencing (RNAseq) were performed by BIOTOOLS (Taipei, Taiwan) based on the manufacturer’s instructions [24]. In brief, the total RNA from zebrafish embryos treated with IS was analyzed by RNAseq using oligo(dT)-labeled magnetic beads. The cDNA was synthesized using fragmented mRNA as a template, and after purification of the beads, end modification, poly A addition at the 3’ end, primer addition, and PCR amplification were performed to construct a complete database. Furthermore, the affected genes were analyzed using Ingenuity Pathway Analysis (IPA, Ingenuity Systems, Redwood City, CA, USA).

### 2.11. Real-Time Quantitative Polymerase Chain Reaction (RT–qPCR)

Total RNA extraction and cDNA synthesis were performed as previously described [22,25]. Briefly, cDNA was synthesized using a high-capacity cDNA reverse transcription kit (Thermo Fisher Scientific-Applied Biosystems, Waltham, MA, USA). qPCR was performed using SYBR Green PCR Master Mix (Applied Biosystems, cat. 4309155) and designed forward and reverse primers. (cox2a-F, 5′-CACTGTTGCCGGACAACTTTCAGA-3′; cox2a-R, 5′-TCCAGCAGTCTGTTTGGTGAAGGA-3′; il1b-F, 5′-GGCTGTGTGTTT GGGAATCT-3′; il1b-R, 5′-TGATAAACCAACCGGGACA-3′, gapdh-F, 5′-GTGGAGTCTACTGGTGTCTTC-3′; gapdh-R, 5′-GTGCAGGAGGCATTGCTTACA-3′). The thermocycling program included 40 continuous cycles of 98 °C for 30 s followed by cooling to 60 °C for 30 s to determine the cycle threshold (Ct). The 2^−^^ΔΔCt^ equation was used to calculate the difference in mRNA expression between the experimental and control groups. StepOne Software v2.2.2 (Applied Biosystems, Waltham, MA, USA) was used to analyze the results.

### 2.12. Statistical Analysis

All data are presented as the mean ± SD (standard deviation). All significant differences were statistically analyzed by Student’s *t* test except for the assessments of embryo survival, hatchability, and renal defects, which were performed using Fisher’s exact test as described by Tang et al. [26]. A *p* value < 0.05 was considered statistically significant.

## 3. Results

### 3.1. Assessment of the Effects of IS on the Survival Rate and Hatchability

**Figure 1:**
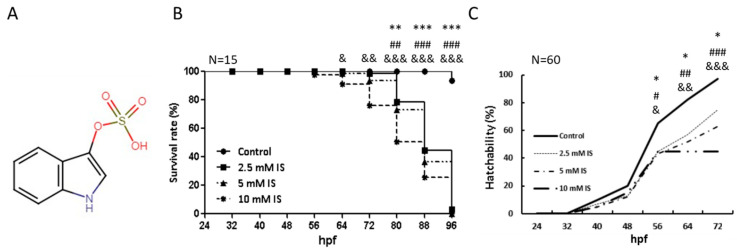
Toxicity profile of IS as indicated by the survival and hatchability of zebrafish embryos (A) Structure of IS. (**B**) Time-course plot of embryo survival in the control group vs. the IS group at 96 h. (C) Hatchability of zebrafish after IS treatment. hpf: hours postfertilization. (**B**,**C**) were statistically analyzed using Fisher’s exact test. * *p* < 0.05, ** *p* < 0.001 and *** *p* < 0.0001, the 2.5 mM IS group compared with the control group. ^#^ *p* < 0.05, ^##^ *p* < 0.001 and ^###^ *p* < 0.0001, the 5 mM IS group compared with the control group. ^&^ *p* < 0.05, ^&&^ *p* < 0.001 and ^&&&^ *p* < 0.0001, the 10 mM IS group compared with the control group. N indicates the embryo number for each group.

Zebrafish have become widely used biological models in chemical toxicity screening tests [12]. Our study showed that the survival rate and hatchability decreased in a dose-dependent manner. Exposure to 2.5 mM IS (Figure 1A) did not affect larval survival at 24 h; the mortality rate significantly increased to 100% at 96 hpf (Figure 1B), suggesting that prolonged exposure to IS affects the survival of zebrafish embryos. To further explore the effects of IS treatment, we counted the embryos that hatched within 72 hpf (Figure 1C). The hatching rates of the IS-treated groups were significantly lower than those of the control group at 56 hpf.

### 3.2. Assessment of the Effect of IS on Cardiac Development

**Figure 2:**
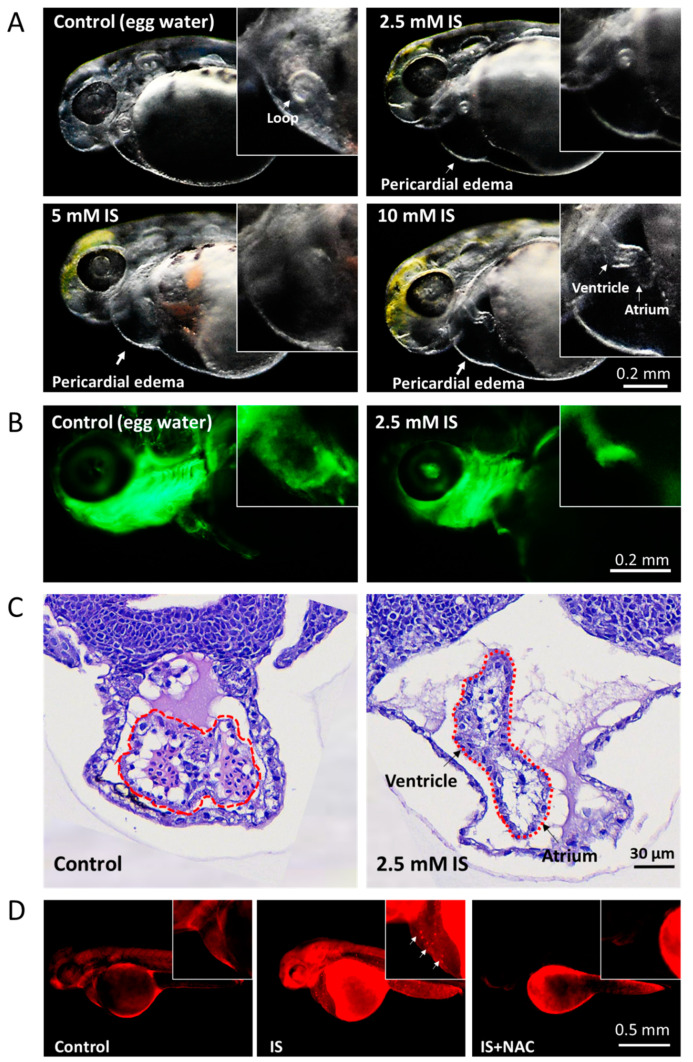
Assessment of the effect of IS on cardiac development in zebrafish embryos. (**A**) Morphological changes in the heart. The results showed that IS exposure causes cardiac dysmorphology and pericardial edema in zebrafish embryos. Furthermore, the atria and ventricles of the heart were elongated and separated. Morphology was assessed visually using a stereomicroscope (Leica MZ10 F). (**B**) *Tg*(*fli1:GFP*) zebrafish embryos were treated with 2.5 mM IS. (**C**) HE staining. Zebrafish embryos treated with 2.5 mM IS were fixed in 10% formalin for one day, immersed in 1% agarose, paraffin-embedded and stained with HE. The stained tissues were observed under a microscope. The dotted lines indicate the developing heart. Compared to the control condition, IS exposure caused elongation of the heart and distal separation of the ventricle and atrium. (**D**) Representative results for ROS production in pericardial tissues using DHE staining. The white arrows indicate the signals where ROS accumulated in the pericardium. Control embryos were soaked in egg water (60 μg/mL sea salt) without IS treatment.

The process of cardiac development includes cell differentiation and morphogenesis as well as elaborate tissue remodeling. Zebrafish have emerged as immensely powerful model organisms for unraveling the genetic, molecular, and cellular mechanisms underlying cardiac development and function [27]. In this study, exposure to IS resulted in pericardial edema and morphological malformations in zebrafish embryos. As shown in the lateral view, the ventricle and atrium developed normally in the control embryos, showing a looping pattern. In IS-treated embryos, we found that the atria and ventricles were elongated and separated (Figure 2A–C). In addition, the results showed that IS exposure affected the heart rates of zebrafish embryos (Video S1).

Previous studies have suggested that IS can cause cytotoxicity by activating NAD(P)H oxidase in cells and decreasing glutathione levels to mediate the release of ROS [28], resulting in oxidization of substances. To determine whether IS induces ROS production and impairs development in the zebrafish model, we further used the probe DHE to evaluate the level of O_2_^•−^ in zebrafish embryos. After IS exposure for 24 h, red fluorescence (DHE) was distributed throughout the zebrafish embryos, especially in the epidermis of the head and body and in the pericardium, suggesting that endogenous ROS production in the zebrafish embryos was increased. Pretreatment with 10 μM *N*-acetylcysteine (NAC), a ROS scavenger, significantly reduced the red fluorescence intensity in zebrafish embryos compared to that in embryos treated with IS alone, confirming that ROS production was induced by IS treatment (Figure 2D).

### 3.3. IS Treatment Led to Kidney Defects in Zebrafish

**Figure 3:**
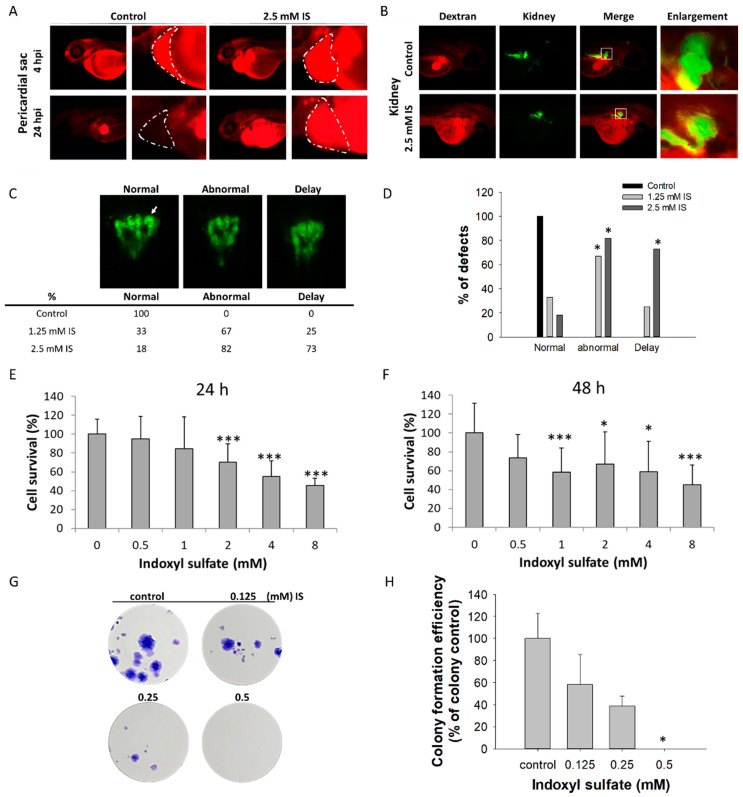
Impact of IS on renal development. The results of the zebrafish assay are shown in (**A**–**D**); those obtained with the HEK293T cell line are shown in (**E**–**H**). After treatment with IS, dextran clearance was reduced, and tubular transport function was disrupted. IS- and vehicle-treated larval zebrafish were injected with 10 kDa rhodamine-labeled dextran at 48 hpf, and the fluorescence of each embryo was measured after 4 and 24 h, respectively. (**A**) Visual comparison of rhodamine-labeled dextran clearance in two individual fish after IS treatment. The impact of IS exposure on renal clearance of dextran was assessed to determine renal function. (**B**) Morphological changes in renal development. ■ Red fluorescence indicates rhodamine-labeled dextran; ■ green fluorescence indicates kidney tissue. (**C**) Results of renal defect assessment. The white arrows indicate perinephric tubules. (**D**) Quantitative results of (**C**) Fisher’s exact test. An MTS-based assay was used to assess the viability of human HEK293T cells treated with IS for (**E**) 24 h and (**F**) 48 h. The results of the colony formation assay are shown in (**G**) HEK293T cells were treated with the indicated concentrations of IS (from 0.125 to 0.5 μM) for 14 days. Afterward, the cells were fixed in 4% paraformaldehyde and stained with Giemsa. (**H**) Quantitative analysis of (**G**) the colony formation assay. * *p* < 0.05, *** *p* < 0.005.

To evaluate the impact of IS on the renal function of zebrafish embryos, the embryos were soaked with 2.5 mM IS at 24 hpf and injected with 50 ng of 10 kDa dextran conjugated with Rhodamine fluorescent dye into the cardiac venous sinus at 48 hpf. These images of individual embryos were taken using a fluorescence microscope immediately after dextran injection, as well as 4 h and 24 h after injection. The fluorescence intensity was measured across the heart, which is primarily a vascular compartment (Figure 3A,B). The nephrotoxic effects of IS exposure on renal development of zebrafish embryos were evaluated according to the methods in Bollig’s study [29]. *Tg(wt1b:GFP)* transgenic zebrafish were used to observe renal development. During renal development, pronephric tubules should develop at 65 hpf. However, our experiments showed that at 72 hpf, the proportions of embryos with abnormal renal development after exposure to 1.25 mM and 2.5 mM IS were 67% and 82%, respectively, while the proportions of embryos with delayed renal development, which was defined by pronephric tubules that were not fully developed after 65 h, were 25% and 73%, respectively (Figure 3C,D). We further evaluated the cytotoxicity of IS using the human embryonic kidney cell line HEK293T, and cell death was observed to be significant in the groups treated with 1 and 2 mM IS after 24 and 48 h (Figure 3E,F). Colony formation assays were performed using IS-treated HEK293T cells (Figure 3G,H), and the results were consistent, suggesting that IS exposure attenuates the proliferation ability of kidney epithelial cells.

### 3.4. IS Induces MAPK Pathway-Associated Gene Expression

**Figure 4:**
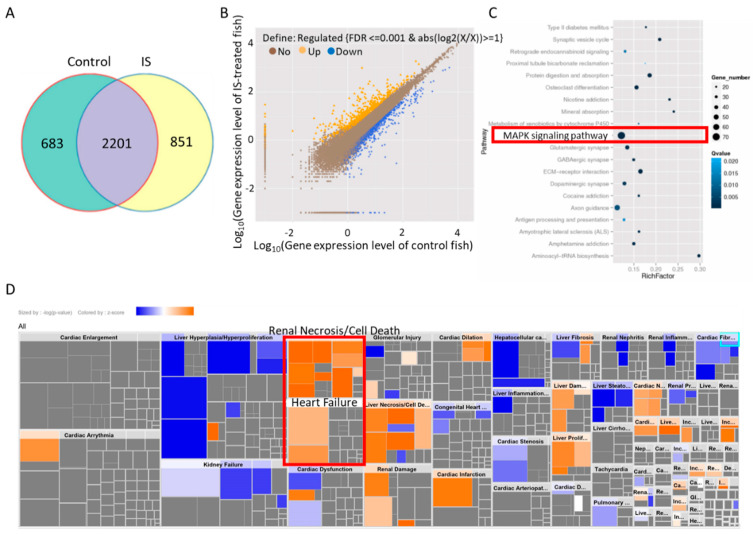
KEGG enrichment and IPA of differentially expressed genes in zebrafish. (**A**) The Venn diagram shows the coexpressed genes between IS-treated and control embryos. (**B**) Scatter plots of all expressed genes between IS-treated and control zebrafish embryos plotted as log_10_ (gene expression level with FDR ≤ 0.001 & abs(log2(X/X)) ≥ 1). (**C**) Scatter plot of KEGG enrichment analysis of the differentially expressed genes between IS-treated and control fish embryos. The red border indicates the candidate MAPK pathway. (**D**) Toxicological functions of the differentially expressed genes related to IS exposure.

After collecting the zebrafish embryos treated with IS, the total RNA was converted into cDNA, which was then analyzed using NGS. The sequences were compared with the reference database for further analysis of the obtained gene expression. IPA and the Kyoto Encyclopedia of Genes and Genomes (KEGG, https://www.genome.jp/kegg/kegg3a.html accessed on 18 November 2021) were used for bioinformatics analysis to predict the genes and candidate pathways. A total of 22201 genes were coexpressed in the IS-treated and control fish, while 683 and 851 were uniquely expressed in the IS and control groups, respectively (Figure 4A). A total of 608 transcripts were upregulated, while 1200 transcripts were downregulated, in response to IS treatment (Figure 4B). Among the top 20 enriched pathways, the MAPK pathway had the greatest gene number, which was more significant than most of the pathways (Figure 4C). We subsequently performed gene set enrichment analysis using IPA software on the differentially expressed genes in IS-treated zebrafish embryos. The results of toxicological function analysis revealed that renal necrosis/cell death and heart failure were activated in response to IS exposure (Figure 4D).

### 3.5. IS Affects Zebrafish Development via the MAPK Pathway

**Figure 5:**
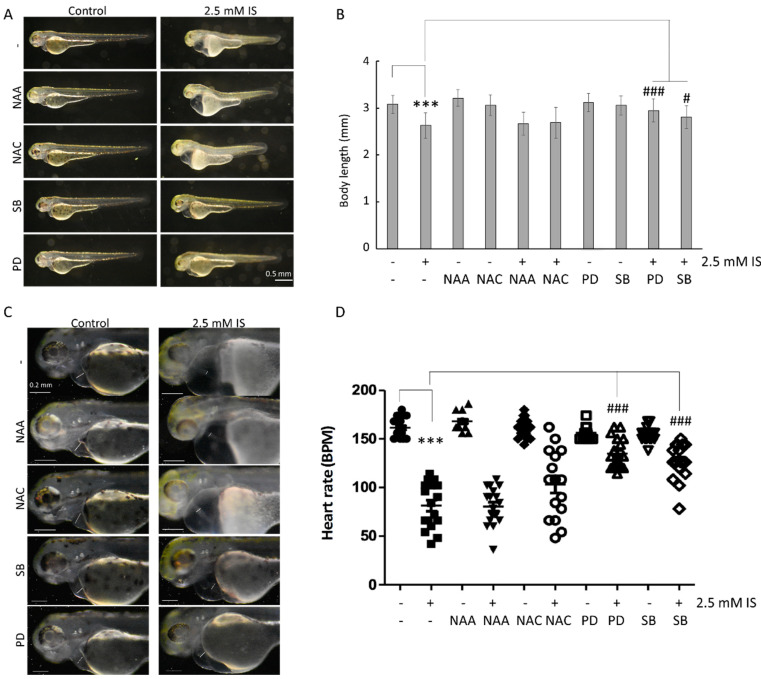
Inhibition of MAPK pathways attenuates the impact of IS on the embryonic development of zebrafish. (**A**) Changes in embryo body length. (**B**) Quantitative results for (**A**). (**C**) Survival analysis. (**D**) Analysis of heart rate. SB: SB203580, a MAPK p38 inhibitor; PD: PD98059, a MAPK ERK inhibitor. NAA was used as a negative control for ROS scavenging. *** *p* < 0.001 compared with the control group. # *p* < 0.05 and ### *p* < 0.001 compared with the group with IS exposure.

Zebrafish embryos exposed to 2.5 mM IS were shorter than control embryos (Figure 5A,B) and exhibited a greater incidence of pericardial edema and axial malformations. Additionally, IS affected the heart rate of zebrafish (Video S1). Zebrafish embryos were also treated with NAC; N-acetyl-alanine (NAA), a NAC analog without ROS-scavenging activity; SB203580, a p38 inhibitor; and PD98059, an ERK inhibitor. The results showed that the inhibition of p38 and ERK reversed the changes in body length (Figure 5A,B) and heart rate (Figure 5C,D) in zebrafish embryos induced by IS exposure.

**Figure 6:**
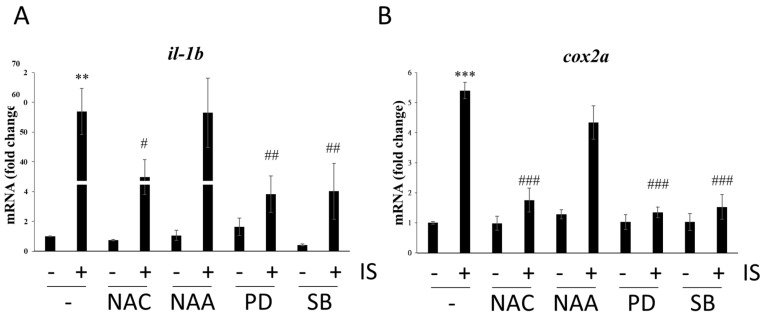
Effects of ROS scavengers and MAPK inhibitors on the mRNA expression of proinflammatory genes. qPCR-based assessment of the mRNA levels of (**A**) *il-1b* and (**B**) *cox2a.* ** *p* < 0.01 and *** *p* < 0.001 compared with the control group. # *p* < 0.05, ## *p* < 0.01 and ### *p* < 0.001 compared with the IS-treated group.

IS exposure has been reported to increase pro-oxidation and pro-inflammatory reactions in a mouse study [30]. In our study, the results showed that both ROS scavenging and blockade of the MAPK pathway significantly reduced the mRNA levels of the proinflammatory genes *il-1b* and *cox2a,* whose expression was induced by IS (Figure 6), suggesting that IS may cause inflammation through oxidative stress and the MAPK pathway.

## 4. Discussion

The 2017 annual report of the United States Renal Data System (USRDS) indicated that people in Taiwan have the highest prevalence and incidence of end-stage renal disease (ESRD) compared to people in other countries [31]. CKD is currently defined by the presence of a glomerular filtration rate (GFR) lower than 60 mL/min/1.73 m^2^ or kidney structural or functional damage for at least three months [32]. Almost no symptoms are noticed in the early stages of CKD, so it is important to understand the high-risk groups for CKD [33]. Diabetes, hypertension, cardiovascular disease, history of acute kidney injury, old age, obesity or metabolic syndrome, long-term use of analgesics, smoking, hyperuricemia, and gout may all be risk factors for CKD. Although hemodialysis technology has advanced, it is still unable to effectively remove all types and sizes of uremic toxins, resulting in complications during dialysis [34]. At present, hemodialysis is the method most commonly used to remove solute molecules. Nephrotoxins can be divided into several categories, namely, small water-soluble molecules (MW < 500 Daltons), medium-sized molecules (middle molecules), and protein-bound molecules [35,36]. IS is classified as a protein-bound molecule.

The uremic toxin IS, which is derived from tryptophan metabolism, is an endotheliotoxin largely involved in the genesis of cardiovascular disorders in CKD patients. In endothelial cells, IS has been shown to promote pro-oxidation, pro-inflammatory, and pro-thrombotic processes that are involved in the dysfunction of endothelial tissues. This endothelial dysfunction could induce altered vascular repair, arteriosclerosis, and thrombosis [2]. Tryptophan consumed in daily food is metabolized into indole by intestinal microbes such as *E. coli* and absorbed into blood circulation [37]; then, the indole is transported to the liver through the blood, and the liver absorbs and metabolizes it into IS [38]. In healthy humans, IS is taken up by organic anion transporter 1 (OAT1) and organic anion transporter 3 (OAT3) on the basolateral membranes of tubular cells in the kidneys [39] and is excreted into the renal tubules to form urine. Finally, it is excreted from the body. However, in CKD patients, the kidneys are unable to filter urinary toxins; thus, the IS is not completely excreted [40]. Moreover, because IS binds to albumin, it cannot be excreted entirely through hemodialysis [3], which causes the patient’s health to deteriorate.

CKD is one of the most common diseases and causes a considerable burden for public health systems in developed countries [32]. Patients with CKD are at increased risk of cardiovascular illness as their renal failure progresses [41]. CKD increases the risk of thrombotic disorders, such as stroke and myocardial infarction, and accelerates atherosclerosis and venous thromboembolism, which could cause ischemic heart diseases, peripheral vascular diseases, and heart failure [42,43,44].

Arinze et al. observed that doses as low as 50–100 μM IS suppressed vasculogenesis, including decreases in the thickness and bifurcation of the intersegmental vessel (ISV) and tail microvasculature in zebrafish embryos [45]; however, it was still unclear whether IS exposure affects the development of zebrafish embryos and the potential underlying mechanism. In our study, we intended to investigate the acute response of zebrafish to exposure to a high dose of IS in a short time, and our results showed that at a concentration of 2.5 mM, IS did not significantly affect the survival rate but did dysregulate both renal and cardiac development in zebrafish embryos.

Under normal conditions, atrial/ventricular loop formation can both be observed in the early stage of cardiac development in zebrafish [18]. However, in the current study, IS treatment caused the embryonic heart to develop edema and deformity, and the atria and ventricles became elongated and separated (Figure 2).

Moreover, a mouse-based model study investigating IS-induced oxidative stress revealed that a significant fluorescence signal of H2DCF-DA (an H_2_O_2_-sensitive fluorescent probe) was detectable in primary mouse peritoneal macrophages after 3 h and 6 h of IS treatment, and the expression of the antioxidant enzyme SOD-2 was reduced [30]. Additionally, another study showed that injection of unilaterally nephrectomized mice with IS significantly increased the levels of the lipid peroxidation indicator malondialdehyde (MDA) in mouse urine and serum. Similarly, significantly decreased levels of antioxidants, including glutathione, SOD-1/SOD-2, and catalase, were detected [46], indicating that IS may induce oxidative stress by reducing endogenous antioxidant levels.

A critical characteristic of CKD is an increase in oxidative stress along with impaired renal function and the associated consequences, such as thrombosis and atherosclerosis [47]. In our study, ROS-sensitive signals could be detected in the whole bodies of zebrafish embryos, especially the pericardial tissues, when the embryos were stained with the ROS-sensitive fluorescent probe DHE. In contrast, the oxidative fluorescence signal in the pericardium was abolished by NAC pretreatment (Figure 2D).

In mouse experiments, IS has been found to not only affect ROS production but also cause activation of MAPK. IS increases platelet activity in mice by increasing ROS-induced activation of p38MAPK [48]. Interestingly, our study showed that inhibiting MAPK attenuated IS-induced body length shortening, abnormal cardiac morphogenesis, and decreased heart rate in zebrafish embryos through both the p38MAPK and ERK pathways (Figure 5), revealing that the results obtained with zebrafish models and mouse models can complement each other.

A study by Marzocco et al. indicated that IS has proinflammatory effects on C57BL/6J mice and increases the expression of *cox-2* and *Bax* [30]. Another study revealed that neuroinflammation [46], serum IL-1 protein levels, and prefrontal cortical tissue IL-1 mRNA levels are elevated in IS-treated mice. There is no direct evidence or data to allow the comparison of transcriptomic data between IS-treated zebrafish and uremic patients. A study comparing single-cell transcriptomics in human and zebrafish oocytes demonstrated concurrently high expression of orthologous genes and similar functional categories in the two organisms [49]. The MAPK pathway did not have the highest enrichment score but presented a low Q value in the KEGG enrichment analysis. We focused on the MAPK pathway because indole metabolites activate the MAPK pathway in human endothelial cells [50,51,52], which provides a direct link to the pathogenesis of cardiovascular disease in humans. Studies have also suggested that MAPK pathways play an important role in the pathogenesis of cardiac and vascular diseases, especially in cardiac hypertrophy and cardiac remodeling after myocardial infarction [53,54]. These pieces of evidence also support our finding of IS toxicity during cardiac development.

In our study, NGS demonstrated that inflammatory genes were upregulated after IS treatment in zebrafish (Figure 4). The qPCR results showed that the inflammation-related genes *il-1b* and* cox2a* were upregulated (Figure 6), which was consistent with the NGS results. A previous study showed that IS promotes inflammation in mice [30], and our results revealed that IS upregulated the expression of inflammation-associated genes (Figure 6). In contrast, either the NAC scavenger or MAPK inhibitors reversed the IS-induced upregulation of proinflammatory factor mRNA expression (Figure 6), suggesting the involvement of ROS and MAPK activation in IS-induced inflammation in zebrafish embryos and disturbance of renal and cardiac development.

## 5. Conclusions

**Figure 7:**
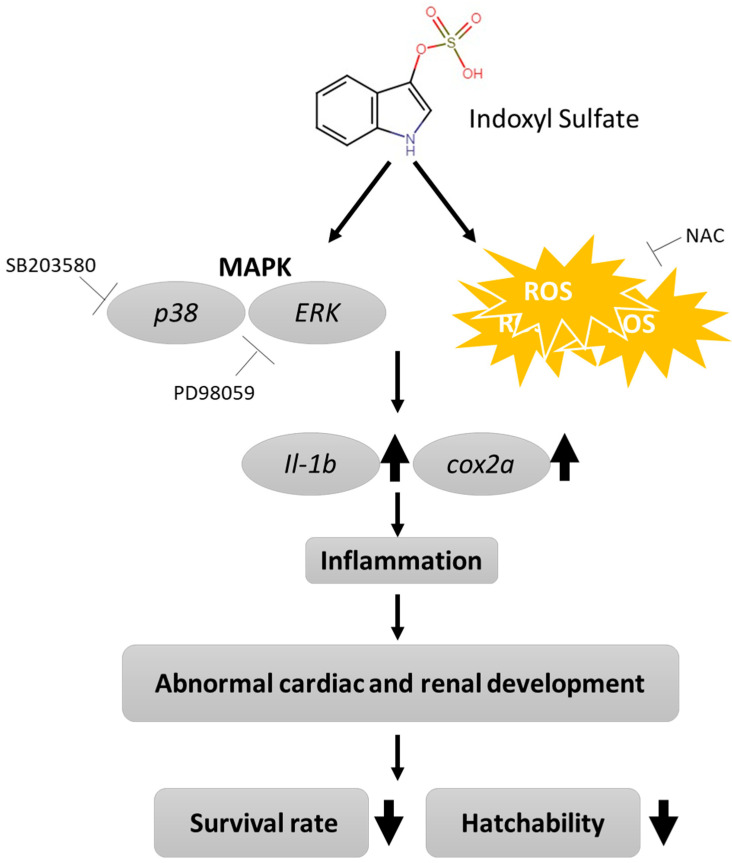
Proposed mechanism of IS-induced morphogenesis and physiological abnormalities during development in zebrafish embryos.

CKD is a strong risk factor for cardiovascular disease, but it is difficult to simultaneously observe the effects of IS on both cardiac and renal development using mouse models alone. Our study demonstrates that IS exposure causes cardiac edema, increased mortality, and moderately shortened body length in zebrafish embryos. The results also suggest that IS disturbs the morphogenesis and functions of the heart and kidneys in zebrafish embryos, providing new evidence of the toxicity of IS to organisms (Figure 7). Importantly, with the advantages of zebrafish in vivo models, we can assess cardiac and renal development and function simultaneously, providing more comprehensive information and allowing studies to be conducted in zebrafish that are complementary to studies conducted in mouse models of cardiac and renal toxicology and injury. Zebrafish models will also benefit high-throughput drug screening for the treatment of both heart- and kidney-associated diseases in the future.

## Data Availability

All of the data are contained within the article and the supplementary materials.

## Supplementary Materials

The following supporting information can be downloaded at: https://www.mdpi.com/article/10.3390/antiox11020400/s1, Video S1: Heart rate of zebrafish embryos after IS treatment.
